# The Peace Celebration, July 19, 1919

**Published:** 1919-07-26

**Authors:** 


### The Hospital

The Workers' Newspaper of Administrative Medicine and Institutional Life, Administration, National Insurance and Health.

No. 1729, Vol. LXVI. SATURDAY, JULY 26, 1919. PRICE TWOPENCE

### THE PEACE CELEBRATION, JULY 19, 1919.

The celebration of Peace in these islands on the 19th instant will be gratefully remembered and treasured by all who were privileged to witness or take part in it. It was probably the greatest, most inspiring and most memorable event which has happened in the history of our country. Memorable because the people everywhere welcomed the opportunity to give heartfelt and continuous expression to their feelings of gratitude to and admiration for the brave men and women to whose prowess, courage, forgetfulness of self, and continuous devotion we owe our freedom and deliverance from worse than death. The unity of sentiment and whole-hearted and popular gratitude to our warriors, surgeons, nurses, and women workers was expressed with unanimous acclaim, so that these tried and trained men of war, from the generals down to the humblest sailor and soldier, and from the King and Queen to the youngest trained nurse and V.A.D., felt the people's welcome and gratitude; and the heart of every one of them was cheered and warmed, stimulated and encouraged. Thus the saviours and the saved were united in sympathy and mutual confidence to an extent and in a manner never exceeded, if it has even been reached in the history of the world.

July 19, 1919, was a great day, great in the splendour of its revelation, the simplicity and magnificence of much connected with the Victory March, and the sense of rejoicing and happiness which everywhere prevailed. The illustrations already published of the many wonderful scenes and pictures presented in the course of the celebrations have created a realisation of the splendour of the procession and its incidents, and the men and women taking part in it. The downpour, which unfortunately came in the late afternoon and evening, was nowhere allowed to interfere with the rejoicing and thanksgiving of myriads of people of both sexes and all ages, who stood for hours in the rain and joined in the great concert. Indeed, it was everywhere the same. The children's festivities in the Royal parks, not only the organised dances, old and country, but those which are dear to the humbler people, were maintained with spirit and persistence everywhere. We publish on pages 434 and 435 remarkable evidence of this fact by reproducing from the *Daily Sketch* an illustration of a multitude of umbrellas, revealing the vast audience in Hyde Park which listened to the concert given by the Imperial Choir of 10,000 voices, accompanied by the Massed Bands of the Brigade of Guards. Most of the immense audience remained standing in the pouring rain for two hours at least.

The unity of the people, and their whole-hearted participation and rejoicing in the completion of Peace was especially manifested in the parks. Here the observant found innumerable family parties, when the morning's attractions were over, grouped under the trees and on the grass, enjoying their lunch. A notable feature was the number of old couples to be seen arm-in-arm, with radiant faces, who rejoiced at having an opportunity to take their part in the Victory Rejoicings. The 19th was the children's day too, for everywhere they were welcomed and considered, given the front place and encouraged to disport and enjoy themselves to their hearts' content. We dwell upon these incidents because they demonstrate in our view the unity of the nation on this day of celebration of the greatest victory of the world, and the upholding of the freedom of its peoples. Are this splendid victory and its fruits, this remarkable unity in patriotic rejoicing, to end with the Peace Celebrations? Surely not, if the happenings on Peace Day continue to possess the solid value which its atmosphere everywhere throughout that day proclaimed.

It has been the habit for years to treat the National Anthem mainly as an indication that some performance or entertainment had terminated. We welcome, therefore, the restoration of "God Save the King" to its proper place and value as the National Anthem. When the King and Queen led the Royal party from Buckingham Palace to the Royal Pavilion in front of the Queen Victoria Memorial, the vast assembly, as Their Majesties appeared, joined voices like one great choir, in singing with heart and conviction the National Anthem. When the ceremony ended, this action was repeated by the vast crowd with renewed enthusiasm as Their Majesties returned to Buckingham Palace. The Mall never before looked so imposing from the Admiralty Arch to the Queen Victoria Memorial as it did on the 19th instant, and the fervour of the people was cheered and heightened by a welcome burst of sunshine.

His Majesty stood on the curved front of the Pavilion; he wore the uniform of a Field-Marshal, and by his side were the Queen and other members of the Royal Family. Immediately behind him stood the Prime Minister, whilst within the enclosure were the members of the Cabinet, statesmen of different political creeds, and representatives of the Allied Powers. When His Majesty received the warriors, and the Admiral and Generals who led them to victory, the scene was not only memorable but great, not only great but grateful to the hearts of warriors and welcomers. What a reception was given to the Navy, to the minesweepers, to members of Queen Alexandra's Nursing Service, and the contingent of the Women's Royal Naval Service, together with men from the brave Mercantile Marine, and the pilots in civilian dress who passed the saluting point waving their hats and cheering the King. The Army too, and the noticeable effect which its coming had on the discharged soldiers in blue, who, by the King's thoughtfulness, were placed in a good position near the pavilion where they could see everything and enjoy! The enthusiasm reached a high point when Sir Douglas Haig approached the Royal Enclosure. The Massed Standards of the Household Cavalry, the Colours of the Brigade of Guards, the Standards and Colours of Cavalry and Infantry, of the Yeomanry and of the Territorial Force stirred the imagination of the people, and the earnestness of Marshal Foch, who came forward by the side of the King, and saluted, moved the hearts of all and raised enthusiasm to the highest point.

Many things happened that we might add to this brief summary of a glorious morning's proceedings; but enough. Everywhere there was evidence of unity and agreement, for every one rejoiced and all were glad. May the sentiments exhibited and expressed on the morning of July 19 during the Victory March enter deeply into the hearts of the people throughout the whole Nation and Empire. May they all retain them with increasing force as the days pass. The very life and expansion and safety and continued existence of our nation and Empire must rest upon the oneness of its peoples and their whole-hearted, united, continuous endeavour to maintain the liberty they have won, the greatness of the position they have attained to, and the glory and respect in which they are held to-day by other nations throughout the civilised world. May all men and women of all ranks and classes, from the humblest to the highest, devote their utmost endeavours and energies to the great tasks which lie before us. Now, assuredly, Nation, Empire, and People must whole-heartedly, unitedly, and continuously work together with a will for the good of all.

